# Top-Down Enrichment Strategy to Co-cultivate Lactic Acid and Lignocellulolytic Bacteria From the *Megathyrsus maximus* Phyllosphere

**DOI:** 10.3389/fmicb.2021.744075

**Published:** 2021-11-02

**Authors:** Laura Díaz-García, Dayanne Chaparro, Hugo Jiménez, Luis Fernando Gómez-Ramírez, Adriana J. Bernal, Esteban Burbano-Erazo, Diego Javier Jiménez

**Affiliations:** ^1^Microbiomes and Bioenergy Research Group, Department of Biological Sciences, Universidad de los Andes, Bogotá, Colombia; ^2^Colombian Corporation for Agricultural Research (Agrosavia), Mosquera, Colombia; ^3^Laboratory of Molecular Interactions of Agricultural Microbes, Department of Biological Sciences, Universidad de los Andes, Bogotá, Colombia

**Keywords:** bacterial consortia, lignocellulose, metagenome-assembled genomes, silage, bioinoculant, *Cellulosimicrobium*

## Abstract

Traditionally, starting inoculants have been applied to improve ensiling of forage used for livestock feed. Here, we aimed to build up a bioinoculant composed of lactic acid-producing and lignocellulolytic bacteria (LB) derived from the *Megathyrsus maximus* (guinea grass) phyllosphere. For this, the dilution-to-stimulation approach was used, including a sequential modification of the starting culture medium [Man, Rogosa, and Sharpe (MRS) broth] by addition of plant biomass (PB) and elimination of labile carbon sources. Along 10 growth-dilution steps (T1–T10), slight differences were observed in terms of bacterial diversity and composition. After the sixth subculture, the consortium started to degrade PB, decreasing its growth rate. The co-existence of *Enterobacteriales* (fast growers and highly abundance), *Actinomycetales*, *Bacillales*, and *Lactobacillales* species was observed at the end of the selection process. However, a significant structural change was noticed when the mixed consortium was cultivated in higher volume (500ml) for 8days, mainly increasing the proportion of *Paenibacillaceae* populations. Interestingly, *Actinomycetales*, *Bacillales*, and *Lactobacillales* respond positively to a pH decrease (4–5), suggesting a relevant role within a further silage process. Moreover, gene-centric metagenomic analysis showed an increase of (hemi)cellulose-degrading enzymes (HDEs) during the enrichment strategy. Reconstruction of metagenome-assembled genomes (MAGs) revealed that *Paenibacillus*, *Cellulosimicrobium*, and *Sphingomonas* appear as key (hemi)cellulolytic members (harboring endo-glucanases/xylanases, arabinofuranosidases, and esterases), whereas *Enterococcus* and *Cellulosimicrobium* have the potential to degrade oligosaccharides, metabolize xylose and might produce lactic acid through the phosphoketolase (PK) pathway. Based on this evidence, we conclude that our innovative top-down strategy enriched a unique bacterial consortium that could be useful in biotechnological applications, including the development/design of a synthetic bioinoculant to improve silage processes.

## Introduction

Ensiling is an ancient technique used to preserve nutrients in roughages (e.g., grass and legumes), generally offered as a ruminant livestock feed (Ávila and Carvalho, 2019). This process is based on spontaneous fermentation, where epiphytic lactic acid bacteria (LAB) metabolize plant-derived sugars, quickly decreasing the pH (between 5 and 4) and preventing silage spoilage (Fabiszewska et al., 2019). *Megathyrsus maximus* Jacq., a fast-growing, leafy perennial grass, is widely used in the tropics as roughage for livestock farming. Due to its high biomass availability (during both the dry and rainy seasons), protein content (~13%) and livestock digestibility (~60%), this grass has great potential for ensiling (Mojica-Rodríguez and Burbano-Erazo, 2020). However, *M. maximus* (guinea grass) like many tropical grasses contains a large amount of fiber, including lignin and structural carbohydrates linked to ferulic acid, preventing its efficient utilization directly in feedlots (Grabber et al., 2009). Ensiling of roughages comprises four phases: (1) microbial depletion of oxygen; (2) lactic acid production in anaerobic conditions (here LAB becomes highly abundant); (3) stabilization; and (4) feedout phase where silage is re-exposed to air (Keshri et al., 2019). The type of epiphytic microbiota depends on the forage crop, and is mainly composed of Gammaproteobacteria (e.g., *Klebsiella*, *Citrobacter*, *Pantoea*, and *Pseudomonas*), Firmicutes (e.g., *Clostridium*, *Bacillus*, and *Paenibacillus*), yeast, and molds, plus a low proportion of LAB (e.g., *Lactobacillus*, *Pediococcus*, *Enterococcus*, *Lactococcus*, *Leuconostoc*, and *Weissella*), actinomycetes and acetic/propionic acid bacteria (Nazar et al., 2021a, b). The phyllosphere of forage could represent an unexplored source of lignocellulolytic microbes that survive in oligotrophic conditions. Regarding ensiling, LAB populations are essential, but they do not exceed 1% of the total epiphytic microbiota (McAllister et al., 2018, Ávila and Carvalho, 2019; Fabiszewska et al., 2019). Therefore, the use of starting homofermentative LAB bioinoculants (e.g., *Lactobacillus* species) and other additives (e.g., organic acids and enzymes) has been used to improve the quality of the silage, thus benefiting animal productivity (Muck et al., 2018; Su et al., 2019). Additionally, (hemi)cellulases, amylases and proteases have also been added to forage at initiation of ensiling to improve the breakdown of plant polymers, providing labile sugars for lactic acid production and increasing the digestibility of plant cell walls (Windle et al., 2014; Addah et al., 2016; Ogunade et al., 2018). Moreover, it has been suggested that the inoculation of bacterial isolates and/or mixed microbial consortia (e.g., LAB plus lignocellulolytic species) with the potential to produce cellulases, arabinofuranosidases, xylosidases, ferulic acid esterases (FAE; EC 3.1.1.73), and cutinases, could enhance the quality of the silage by increasing the availability of carbohydrates with lower complexity (Muck et al., 2018; Tarraran and Mazzoli, 2018; Bonaldi et al., 2021). FAE has been recently reported in LAB, and its use may show a potential improvement on neutral detergent fiber (Xie et al., 2021).

The design and/or selection of microbial consortia with a desired structure/function can be accomplished by two strategies. In the top-down approach, microbial communities from nature are selected in a specific culture medium that enables the survival of the fittest in sequential transfers (Jiménez et al., 2014a; Díaz-García et al., 2021). On the other hand, in the bottom-up approach, the design of the consortium is carried out by mixing different populations of microbial isolates in specific proportions and compositions (Jiménez et al., 2018, 2020). Interestingly, a synthetic consortium composed of LAB and a cellulolytic fungus has been previously reported (Shahab et al., 2018). In this mutualistic guild, *Trichoderma reesei* provides the soluble saccharides (by depolymerization of plant polysaccharides), whereas *Lactobacillus pentosus* utilize them for the production of lactic and acetic acid. Based on these facts, we hypothesize that the selection of consortia composed of LAB and lignocellulolytic bacteria (LB), derived from the epiphytic community of forage biomass, and could be the starting point to design a synthetic bioinoculant that preserves the quality of the plant biomass (PB) during the ensiling process. In addition, the discovery of microbes or a set of mutualistic species that can produce lactic acid directly from PB is interesting from a biotechnological perspective (Tarraran and Mazzoli, 2018). Thus, in this study, we aimed to co-cultivate LAB and LB derived from the *M. maximus* cv. Agrosavia Sabanera phyllosphere by using a top-down approach (i.e., the dilution-to-stimulation strategy in which easy-to-consume carbon sources are eliminated and growth on the desired substrate is stimulated). During and after the selection process, different modifications of culture conditions were done. Additionally, bacterial 16S rRNA gene and whole metagenome sequencing, including the reconstruction of metagenome assembled genomes (MAGs), was performed to disentangle taxonomic and functional profiles of the obtained consortia. Based on this enrichment and metagenomic analysis, we report here the genomic capacity of a (hemi)cellulolytic *Cellulosimicrobium*-related species (an actinobacterium) to produce lactic acid from plant-derived xylose.

## Materials and Methods

### Enrichment Strategy to Co-cultivated Lactic Acid and Lignocellulolytic Bacteria

**Figure 1:**
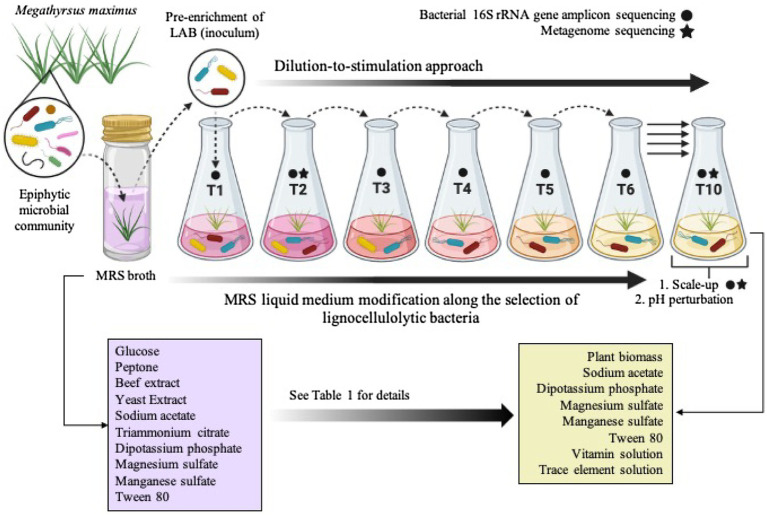
Schematic representation of the top-down enrichment strategy by using the dilution-to-stimulation approach. During the selection process, the liquid Man, Rogosa, and Sharpe (MRS) medium, which is specific for lactic acid bacteria (LAB), was modified as shown in Table 1 in order to select lignocellulolytic bacteria (LB) from the *Megathyrsus maximus* phyllosphere. The scale-up and pH perturbation were carried out after transfer 10 (T10). Created with BioRender.com.

The Guinea grass *M. maximus*-associated epiphytic microbial community was the source of inoculum for selection of LAB and LB populations. Firstly, this grass was cut in 1cm^2^ pieces. Then, in aseptic conditions, 10 fragments were inoculated in 10ml of Man, Rogosa, and Sharpe (MRS) broth (Merck, Darmstadt, Germany) and incubated at 39°C for 24h. After microbial growth, 1ml of the culture was inoculated in 9ml of MRS broth and incubated to the above conditions. This latter procedure was carried out to pre-enrich LAB populations by growth of the community in medium designed to enrich growth of LAB. Subsequently, 25μl of the pre-enriched culture were inoculated in 100ml-flasks with 25ml of MRS-modified broth (i.e., without glucose) containing 1% lignocellulosic substrate (*M. maximus* cv. Agrosavia Sabanera added as a carbon source; T1; Figure 1; Table 1), vitamin and trace elements solution (Jiménez et al., 2014b). Previously, the lignocellulosic substrate (supplied by Agrosavia) was knife-milled through a 1mm screen and washed twice with distilled water and ethanol 70% (v/v). The grass was collected at regrowth age of 30days in a sub-region of the Colombian dry Caribbean (Motilonia RC, Cesar) with an elevation of 103 masl. The culture flasks were incubated at 28°C under shaking conditions (130rpm). After 4days of incubation, aliquots (25μl) of microbial suspension were transferred to 25ml of fresh liquid medium containing 1% plant biomass (T2), following the dilution-to-stimulation approach (Jiménez et al., 2017). Two negative controls were also set up: (A) microbial inoculum in liquid modified MRS (without plant biomass and glucose) and (B) uninoculated liquid modified MRS (containing plant biomass and without glucose). During the growth-dilution transfers, liquid culture medium was sequentially modified (Table 1) to avoid the assimilation of labile carbon sources (e.g., ammonium citrate, yeast, and/or beef extract), increasing the selection for LB populations. Thus, these modifications were done until no growth in negative control A was observed (Figure 1; Table 1). At the end of each transfer cultures were filtered and the remain substrate was dried for 24h at 45°C to calculate the percentage of weight loss, following the formula proposed by de Lima Brossi et al. (2016). Bacterial growth was determined using optical density (OD) at 600nm and the number of viable cells (CFU/ml) was quantified by plate counting in R2A and MRS agar (Merck, Darmstadt, Germany). Moreover, samples from transfer 10 (T10) were conserved in 20% of glycerol at −20°C. ANOVA and *post hoc* Tukey-Kramer test were performed using the software R (R Core Team, 2008) to evaluate the significant difference in substrate consumption and bacterial growth along the transfers, with a CI of 99% (*α*=0.01).

**Table 1:** Scheme of MRS broth modification during the enrichment strategy (from T1 to T6–T10).

MRS broth components (w/v)	Modification of MRS broth along the sequential transfers					
	T1	T2	T3	T4	T5	T6–T10
2% Glucose	1% PB	1% PB	1% PB	1% PB	1% PB	1% PB
1% Peptone	O	0.1%	0.01%	X	X	X
0.8% Beef extract	O	0.08%	0.008%	X	X	X
0.5% Sodium acetate	O	O	O	0.25%	0.25%	0.125%
0.4% Yeast extract	O	0.04%	0.004%	X	X	X
0.2% Triammonium citrate	O	O	O	0.1%	0.1%	X
0.2% Dipotassium phosphate	O	O	O	O	0.1%	0.1%
0.02% Magnesium sulfate	O	O	O	O	O	O
0.02% Manganese sulfate	O	O	O	O	O	O
45μl/100ml of Tween 80	O	O	O	O	22.5μl/100ml	22.5μl/100ml

The table shows the composition of modified medium for each enrichment step; all values are % (w/v) except for Tween 80 which is a volume ratio. (O) means the same percentage used in original MRS broth. (X) means the absence of this component within the modified culture medium. At T1, we replaced 2% glucose to 1% of PB and maintained it the same concentration in the subsequent transfers.

### Growth of the Bacterial Consortium at Different Volume and pH Values

To increase the bacterial biomass, the mixed culture after T10 was transferred to a final volume of 500ml. We carried out this procedure because this consortium was used as a starting bioinoculant in a silage of tropical forages in field experiments (data not shown). The scale-up growth process began with a pre-inoculum from T10 that was cultivated in 25ml of liquid medium (eight replicates), as previously described. Two of these replicates were mixed to produce an inoculum of 50ml. To ensure a high bacterial density, four 2L-flasks with 500ml of fresh medium (same composition as T10) were inoculated with the former 50ml and incubated at 28°C for 8days. Finally, four replicates were mixed and filtered to produce 2L of bacterial culture. Moreover, the consortium obtained at T10 was cultivated (i.e., perturbed) on the modified MRS medium adjusted to different pH values (4, 5, and unmodified pH 6.2) at 28°C for 4days, with shaking at 130rpm (in two sequential transfers). Number of viable cells (CFU/ml) was quantified by plate counting in R2A and MRS agar (Merck, Darmstadt, Germany). OD at 600nm and weight loss of the substrate were calculated with the parameters described above. After microbial growth in different pH values, the secreted fractions of the cultures were tested for the presence of plant polymers-degrading endo-enzymes using a set of six chromogenic polysaccharide hydrogels (CPH; i.e., 2-HE-cellulose, carboxymethylcellulose, arabinoxylan, xylan, xyloglucan, and glucomannan) and two insoluble biomass substrates (ICB; i.e., wheat straw and sugarcane bagasse; Kračun et al., 2015). The semi-quantification of the enzymatic activities was measured as reported Díaz-García et al. (2021) and following the instructions of the manufacturer (GlycoSpot IVS, Farum, Denmark). ANOVA and *post hoc* Tukey-Kramer test were performed using the software R (R Core Team, 2008) to evaluate significant differences in growth and enzyme activities, with a CI of 99% (*α*=0.01).

### Total DNA Extraction and 16S rRNA Gene Amplicon Sequencing

Total microbial DNA was extracted using the DNeasy UltraClean Microbial Kit (Qiagen, Hilden, Germany) according to the instructions of the manufacturer. For the 16S rRNA gene analysis, triplicate samples from the dilution-to-stimulation approach (T1, T2, T3, T4, T5, T6, and T10), after the scale-up process (S) and the pH perturbation (4, 5, and unmodified) were sequenced using the Illumina MiSeq technology (300bp pair-end reads). For each sample, a library of 16S rRNA gene amplicons (hypervariable region V3–V4) was prepared using the primers 341F and 785R (Klindworth et al., 2013). Bioinformatic analysis was done using the software Quantitative Insights Into Microbial Ecology (QIIME2; Bolyen et al., 2019). VSEARCH (Rognes et al., 2016) was used to join raw pair-end reads. Quality control and read correction to obtain amplicon sequence variants (ASVs) were done using Deblur pipeline (Amir et al., 2017). Rarefaction was performed (5,205 and 5,490 reads for the enrichment steps and the pH perturbation samples, respectively) per sample to calculate alpha and beta diversity (including Shannon’s index and weighted UniFrac distances), and for comparisons between samples (e.g., relative abundance of ASVs). MAFFT (Katoh and Standley, 2013) was used to construct multiple alignments of ASVs. A phylogenetic tree was built using FasTree (Price et al., 2009) based on MAFFT alignment. Taxonomic affiliation of ASVs was done using SILVA rRNA gene curated database (Quast et al., 2013).

### Metagenome Sequencing and Gene-Centric Analysis

Based on the 16S rRNA sequencing results, consortia T2, T10, and S (in duplicate) were subjected to Illumina MiSeq technology sequencing (300bp paired-end reads) in order to determine taxonomy profiles and metabolic potential. After whole-metagenome sequencing, the FastQ files were uploaded in the MG-RAST server (Meyer et al., 2008). Overlapping sequence pairs were matched, and non-overlapping reads retained as individual reads, after which, dereplication was performed. Duplicate read-based inferred SE estimation and quality trimming (phred score<20) used default settings. Gene predictions were done using the FragGeneScan software, and subsequently, the proteins were annotated based on BLASTX searches against the RefSeq and KEGG databases using an *e*-value cutoff of 1e-15, a minimum alignment length of 50 amino acids, and a minimum identity of 50% (Jiménez et al., 2016). Data from MG-RAST annotation were statistically analyzed using the STAMP package (Parks and Beiko, 2010). Moreover, to evaluate the relative abundance of reads per selected enzyme-encoding gene, the counts were normalized to hits, or unique matches, per million reads, thereby accounting for differences in metagenome sizes. Nine genes involved in heterolactic and/or homolactic fermentation of xylose (Tarraran and Mazzoli, 2018) and 10 genes involved in lignocellulose degradation (Jiménez et al., 2014a; Díaz-García et al., 2021) were analyzed. Heat maps were constructed in the web server Heatmapper using row-Z score for each enzyme (Babicki et al., 2016).

### Reconstruction and Analysis of Metagenome-Assembled Genomes

For the assembly of MAGs, raw sequence data (FastQ files) from T2, T10, and S samples were initially trimmed using the Sickle tool v1.33 with default parameters (available at https://github.com/najoshi/sickle). To avoid misleading results from subsequent binning analysis, carp artifacts were detected. For this, trimmed sequences were filtered against adapter and non-authentic primer sequences originating from the Illumina library preparation (Marter et al., 2021). Metagenome assembly was done using MEGAHIT v1.2.7 (Li et al., 2015) with default parameters. Concoct v1.1.0 (Alneberg et al., 2014), Bowtie v2.3.5 (Langmead and Salzberg, 2012), MetaBAT v2.12.1 (Kang et al., 2015), and MaxBin v2.2.6 (Wu et al., 2016) were used to bin the assembled sequences with default parameters. Binned contigs obtained were subsequently analyzed using DAS tool v1.1.2 (Sieber et al., 2018). To assess the completeness and contamination of the resulting bins, CheckM v1.0.13 was used with the lineage_wf option (Parks et al., 2015). The MAGs were structurally annotated and curated based on rRNAs genes (e.g., 5S, 16S, and 23S) and *rpoB* gene using DFAST v1.2.6 (Tanizawa et al., 2018). Taxonomic assignment was performed using GTDB v1.4.1 (Parks et al., 2020). Functional annotation was done using the RAST (Aziz et al., 2008) and dbCAN webservers (Yin et al., 2012). Moreover, 24 encoding-genes involved in hetero- or homo-fermentation of pentoses and hexoses (Hatti-Kaul et al., 2018; Tarraran and Mazzoli, 2018) were searched within the MAGs.

## Results

### Enrichment Strategy to Co-cultivate Lactic Acid and Lignocellulolytic Bacteria

**Figure 2:**
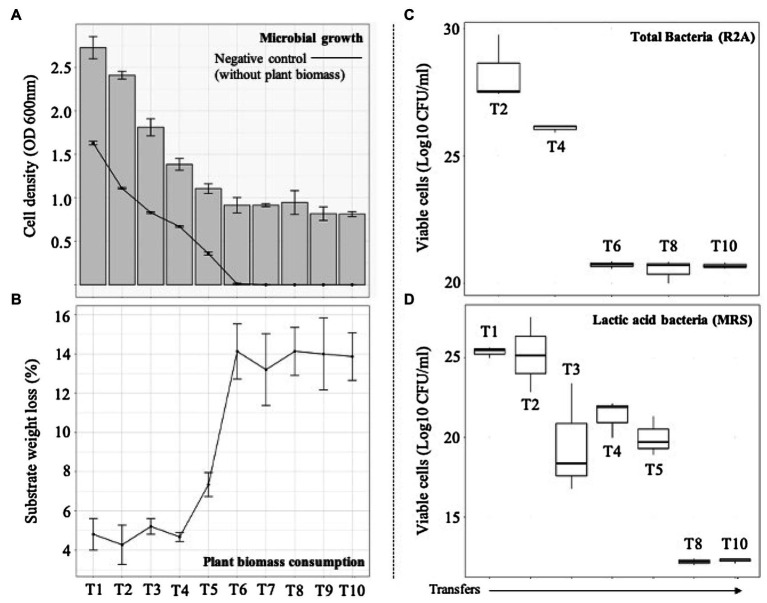
Microbial growth and plant biomass (PB) degradation values after each sequential growth-dilution steps (T1–T10). **(A)** Bars show cell density [optical density (OD) at 600nm] and the line shows cell density in the control medium containing no PB. **(B)** Average substrate weight loss (percent). Error bars represent SDs from three biological replicates. Viable cells (Log10 CFU/ml) of **(C)** total and **(D)** acid lactic bacteria quantified in agar R2A and MRS, respectively.

The selection of consortia was carried out by the dilution-to-stimulation approach using the epiphytic microbial community of *M. maximus* as starting inoculum (Figure 1). In the first stage of selection, we aimed to increase the populations of LAB by growing the phyllosphere-derived community in MRS broth. This pre-enrichment was used to inoculate the first transfer (T1), where glucose was removed and 1% (w/v) *M. maximus* was added as a carbon source. After this stage, increase of LB populations was the main goal. For this purpose, liquid MRS medium was sequentially modified from T2 to T6–T10, removing peptone, yeast extract, beef extract, triammonium citrate, and decreasing the concentration of sodium acetate, dipotassium phosphate, and tween 80 (Table 1). These modifications were carried out until microbial cell growth (measured by OD at 600nm) in the negative control (i.e., without plant biomass) was not observed, indicating that the consortium started to consume the plant polymers (Figure 2A). After T6, the selected consortium was able to consume ~14% of plant biomass and significant differences (value of *p*<0.01) in this capacity were evidenced compared to T5 (Figure 2B). Moreover, progressive reduction of bacterial growth rate was observed from T1 to T6, suggesting that labile carbon sources were depleted due to the liquid medium modification (Figures 2A,C). LAB populations were abundant at T1–T2. However, they decreased along the enrichment strategy (Figure 2D). At the end of the selective process (T10), around 10^9^ and 10^5^CFU/ml of total and LAB populations were obtained, respectively (Figures 2C,D).

### Bacterial Diversity and Composition Along the Enrichment Strategy

**Figure 3:**
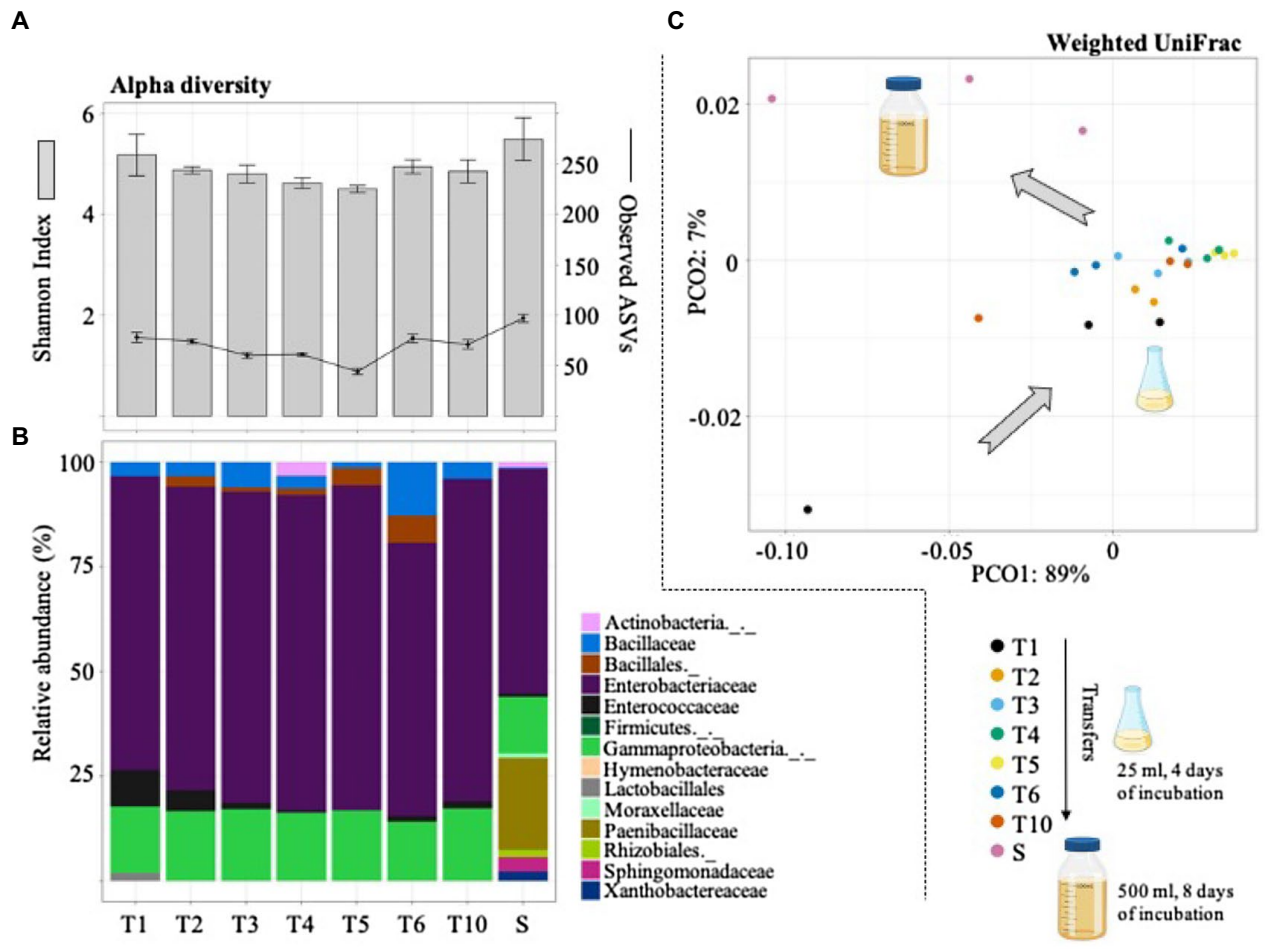
Diversity (alpha and beta) and structural composition of the selected consortia during the sequential growth-dilution steps (T1–T6 and T10) and after its cultivation in a higher volume (i.e., scale-up growth process; S). **(A)** Observed amplicon sequencing variants (ASVs) and Shannon index values. Error bars represent SDs from three biological replicates. **(B)** Relative abundance (%) of taxa (family or higher level) detected using the SILVA database. **(C)** PCoA plot based on weighted UniFrac distances that shows the bacterial community similarity between the sequential transfers and the scale-up growth process (S). Icons were obtained in BioRender.com.

The bacterial community changes during the selective process were evaluated by 16S rRNA gene sequencing. In total, this analysis generated 1,623 ASVs and ~1.2Gbp of high-quality reads. The results indicated that bacterial diversity slightly decreased from T1 to T5. However, the number of observed ASVs increased from T5 (44±3.0) to T6 (77±4.1) and T10 (73±4.5). Interestingly, after the scale-up process (to 500ml of culture volume) the number of ASVs increased to 99±4.0 (Figure 3A). The taxonomic affiliation of the 16S rRNA data revealed that *Gammaproteobacteria* and *Bacillales* species were mostly selected during the enrichment strategy. Regarding LAB populations, species belonging to the *Enterococcaceae* family were observed at T1–T2 (~10% of the total community; Figure 3B). They remained in low abundance in the final selected consortium (i.e., T10). Modifications in the MRS liquid medium did not greatly affect the bacterial structural composition between T2 and T10. Remarkably, the final consortium (i.e., T10) showed a significant change after its cultivation in a higher volume (i.e., 500ml; Figure 3C). This scale-up process (denominated S in Figure 3) mostly increased the relative abundance of *Paenibacillaceae*, *Sphingomonadaceae*, and *Xanthobactereaceae*, well known lignocellulolytic families (Figure 3B). At the end, the mixed bioinoculant (S), used in the field silage experiment, contained a higher proportion of LB populations and lower proportion of LAB (mostly species from *Enterococcaceae* family).

### Gene-Centric Metagenomic Analysis: Taxonomy and Function Within Consortia

**Figure 4:**
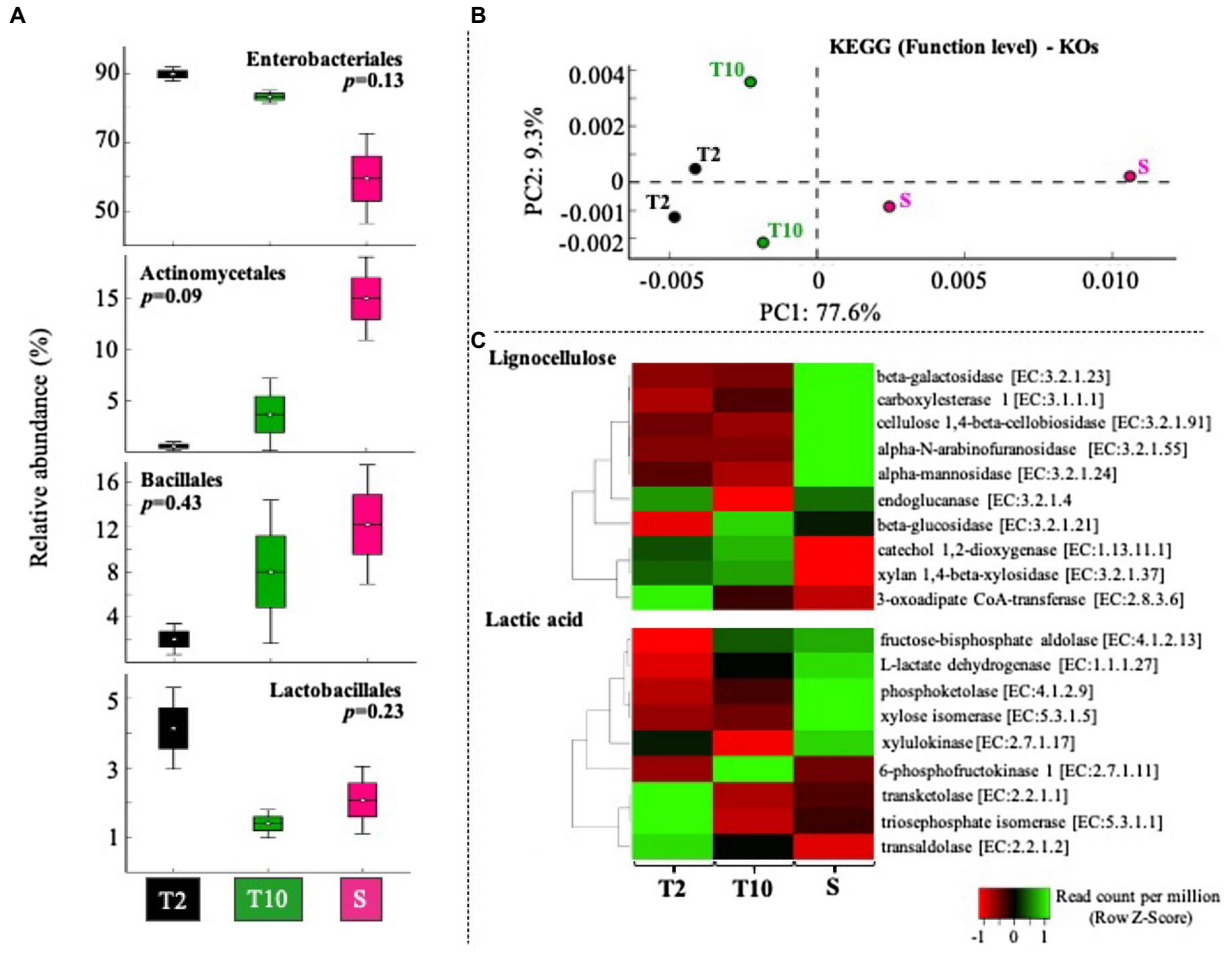
Gene-centric metagenomic analysis of selected consortia after transfer T2, T10, and the scale-up growth process (S). **(A)** Relative proportion (%) of the most abundant orders (*Enterobacteriales*>*Actinomycetales*>*Bacillales*>*Lactobacillales*). Sequences taxonomic assignment was carried out by using RefSeq database. **(B)** Clustering based on functional assignment (KEGG databases) of annotated sequences at KOs level. **(C)** Heat map of normalized abundance values (Row Z score) obtained using the number of sequences annotated within 10 enzyme-encoding genes involved in lignocellulose degradation and nine enzyme-encoding genes involved in heterolactic and/or homolactic fermentation of xylose.

The mixed consortia obtained at T2, T10, and S was subjected to a whole-metagenomic sequencing and analysis. Approximately 1.3, 0.9, and 3.0Gbp were obtained in T2, T10, and S, respectively, with a read average size of 300bp. Based on the taxonomic affiliation of reads, four bacterial classes were highly abundant. In this regard, *Enterobacteriales* and *Lactobacillales* decreased from T2 to T10 and S, while *Actinomycetales* and *Bacillales* increased from T2 to T10 and S (Figure 4A). *Enterobacteriales* species were the predominant taxa (between 50 and 90% of total affiliated sequences) within consortia. At T10 and S, the relative abundance of *Lactobacillales* was lower than at T2, decreasing from about 4% to between 1 and 3% (Figure 4A). Regarding specific members of LAB, *Enterococcus* sp. was the most abundant genus (between 1 and 5% relative abundance) in T2, T10, and S. Other genera, like *Lactobacillus* sp., *Leuconstoc* sp., *Pediococcus* sp., and *Weissella* sp. were found in very low abundance (less than 0.15%) in all the stages of selection, although their populations increased in S with respect to T10 (Supplementary Figure S1). Regarding fungal communities, metagenome data revealed that they are found in very low relative abundance (less than 0.001%). Based on the function (KO level), consortia T2 and T10 showed a similar profile, compared with the consortium obtained after scale-up (S) process (Figure 4B). Moreover, the results showed that nine of 10 genes involved in lignocellulose transformation were enriched from T2 to T10 and S (Figure 4C). Regarding genes relevant to lactic acid production, we observed that genes encoding xylose dissimilation activities [e.g., transketolase (TK) and transaldolase (TA)] were highly abundant at T2, whereas others [e.g., 6-phosphofructokinase and L-lactate dehydrogenase (LDH)] were highly abundant at T10 and S (Figure 4C).

### Response of the Mixed Microbial Consortium in a pH Gradient

**Figure 5:**
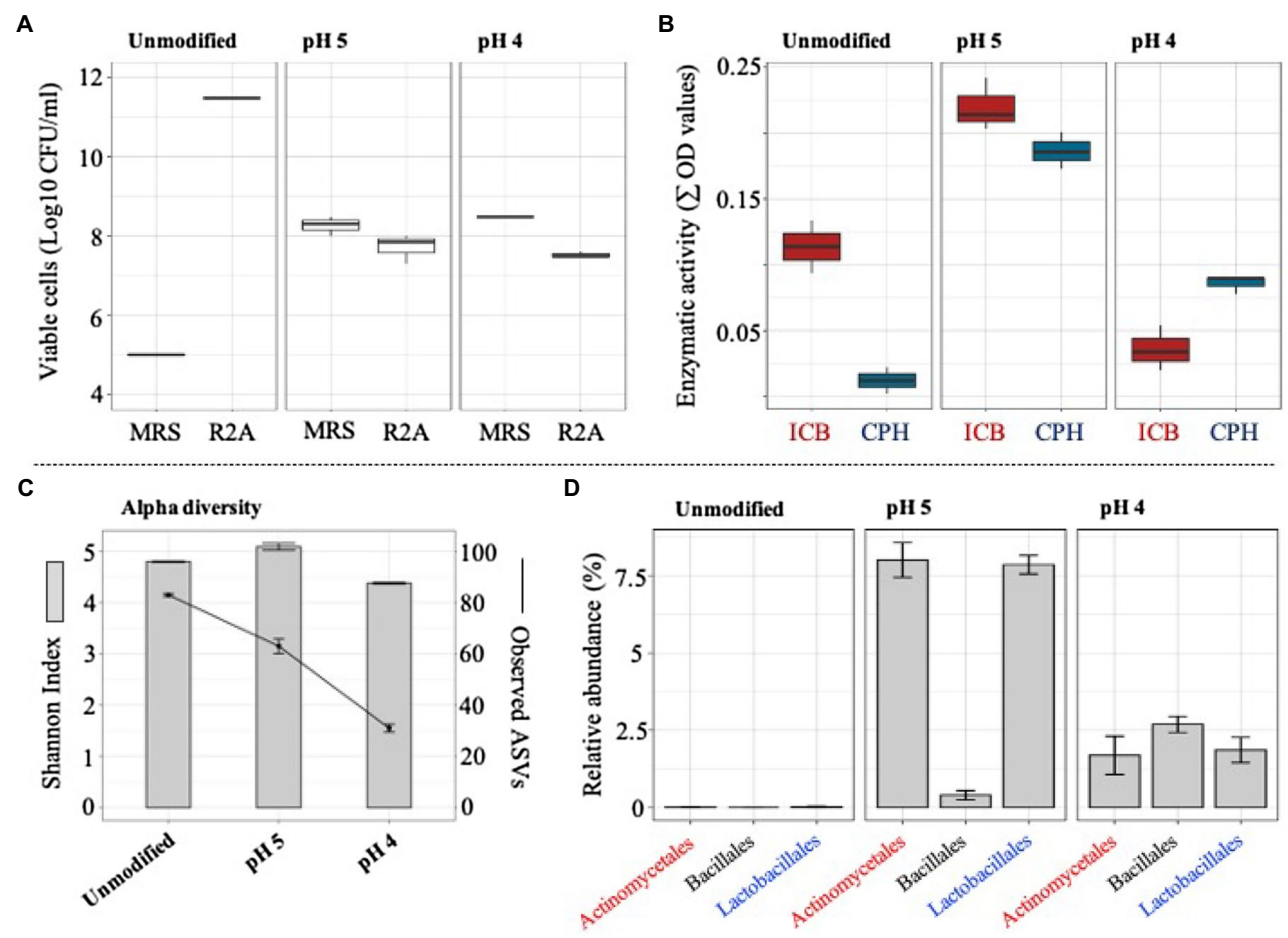
Microbial growth, enzymatic activity, and structural composition of selected consortium (at T10) after its cultivation in lower pH values (unmodified, 5 and 4). **(A)** Viable cells (Log10 CFU/ml) of total and acid lactic bacteria quantified in agar R2A and MRS, respectively. **(B)** Secreted endo-enzymatic activities (∑ OD values) on six chromogenic polysaccharide hydrogels (CPH) and two insoluble biomass substrates (ICB) after T10 consortium growth on unmodified pH, pH 5, and pH 4. **(C)** Observed ASVs and Shannon index values. **(D)** Relative proportion of taxa (class) that increase their abundance at pH 5 and pH 4 (16S rRNA data). Error bars represent SDs from three biological replicates.

The ability of the selected consortium (i.e., T10) to grow in acidic silage conditions was evaluated by modifying the initial pH of the liquid medium. At pH 4, a significant (value of *p*<0.01) increase of LAB was evidenced, showing a viable population density of around 4×10^8^ CFU/ml. In contrast, total bacterial cells decreased at pH 4 and pH 5, compared to the unmodified liquid medium (pH around 6.2; Figure 5A). After the perturbation at different pH values, the metasecretome of the consortium was evaluated for the presence of plant polymer-degrading endo-enzymes. The highest and significant (value of *p*<0.01) enzymatic activities on a set of CPHs and ICBs were obtained in the metasecretome of the mixed consortium after growth at pH 5 (Figure 5B; Supplementary Figure S2). At this pH, the plant biomass consumption was 17.8±0.6%. Additionally, 16S rRNA gene sequencing analysis were carried out after the perturbation at different pH values. On average, 104 ASVs were generated from 50Mbp of high-quality reads. Alpha diversity results demonstrated a decrease of observed ASVs in the pH gradient, reaching a value of 31±1.0 at pH 4 (Figure 5C). Interestingly, a significant (value of *p*<0.001) increase in the relative abundance of *Actinomycetales* and *Lactobacillales* orders was observed at pH 5 (Figure 5D). However, the highest abundant order was *Enterobacteriales* (Supplementary Figure S3).

### Metagenome-Assembled Genomes Obtained From the Mixed Consortia

A total of 2, 3, and 9 high quality MAGs (>90% of completeness and <3.5% of contamination) were reconstructed from the T2, T10, and S metagenomes, respectively (Table 2; Supplementary Table S1). The genome lengths ranged from 2.28 to 5.93Mb. At T2 and T10, MAGs belong to *Cellulosimicrobium funkei* (Bin7_T2 and Bin8_T10) and *Klebsiella aerogenes* (Bin25_T2 and Bin2_T10) were obtained. Interestingly, the data revealed that three different MAGs from *Enterococcus* species were obtained. At T2, a single genome associated to *Enterococcus faecalis*, while in both T10 and S, two genomes associated to *Enterococcus casseliflavus*. Functional annotation of all reconstructed MAGs showed that Bin7_T2, Bin8_T10, Bin3_T10, Bin11_S, Bin6_S, Bin002_S, and Bin8_S contain more than nine genes-encoding glycosyl hydrolases (GHs) from CAZy families involved in degradation of (hemi)cellulose (Table 2). Moreover, Bin3_T10 and Bin6_S affiliated to *E. casseliflavus* harbor more than 20 GHs involved in transformation of oligosaccharides (e.g., GH1, GH2, and GH3). In addition, two LDHs encoding-genes were found in those two MAGs (Table 2; Supplementary Table S2).

**Table 2:** Features of metagenome assembled genome (MAGs) reconstructed from T2, T10, and S consortia.

Transfer	Bin ID	Completeness (%)	Contamination (%)	# contigs	Genome size (Mb)	Probable taxa^*^	Total # CAZymes	ODE (GHs)	HDE (GHs)	CE1	LDHs
T2	Bin7_T2	80.35	1.45	905	3.42	*Cellulosimicrobium funkei*	75	11	10	1	2
T2	Bin2_T2	99.63	0.37	167	2.78	*Enterococcus faecalis*	114	11	0	0	2
T2	Bin25_T2	98.5	2.33	186	5.14	*Klebsiella aerogenes*	119	12	3	2	2
T10	Bin3_T10	71.84	0.31	733	2.28	*Enterococcus casseliflavus*	119	26	14	0	2
T10	Bin8_T10	98.84	1.25	327	4.32	*Cellulosimicrobium funkei*	131	12	10	2	2
T10	Bin4_T10	92.82	0.85	1,006	4.71	*Bacillus paranthracis*	80	2	0	0	2
T10	Bin2_T10	99.92	1.2	101	5.05	*Klebsiella aerogenes*	120	12	3	0	2
S	Bin37_S	95.11	2.62	313	3.06	*Aeromicrobium camelliae*	45	0	1	0	0
S	Bin15_S	98.83	0.34	36	5.06	*Tardiphaga* sp.	79	2	0	1	0
S	Bin11_S	97.84	0.28	143	3.78	*Sphingomonas* sp.	101	10	15	1	0
S	Bin44_S	89.15	0.91	574	4.05	*Aureimonas* sp.	52	0	0	1	1
S	Bin4_S	91.47	6.2	896	4.35	*Brevibacillus borstelensis*	41	1	0	0	0
S	Bin6_S	98.4	3.11	262	3.32	*Enterococcus casseliflavus*	154	34	16	1	2
S	Bin18_S	98.64	2.03	96	5.73	*Brevibacillus* sp.	62	2	1	0	0
S	Bin2_S	94.9	0.2	113	4.82	*Klebsiella aerogenes*	109	12	3	0	2
S	Bin002_S	99.42	2.41	131	4.41	*Cellulosimicrobium funkei*	131	12	10	2	2
S	Bin8_S	98.84	0.23	184	5.93	*Paenibacillus nanensis*	242	24	34	0	0

* Taxa were assigned based on rpoB blast and average nucleotide identity criteria (Parks et al., 2020).

ODE, oligosaccharide-degrading enzymes; HDE, (hemi)cellulose-degrading enzymes; GHs, glycosyl hydrolases; CE, carbohydrate esterases; and LDHs, lactate dehydrogenases (EC 1.1.1.27 and EC 1.1.1.28).

### Analysis of MAGs With the Potential to Produce Lactic Acid

**Figure 6:**
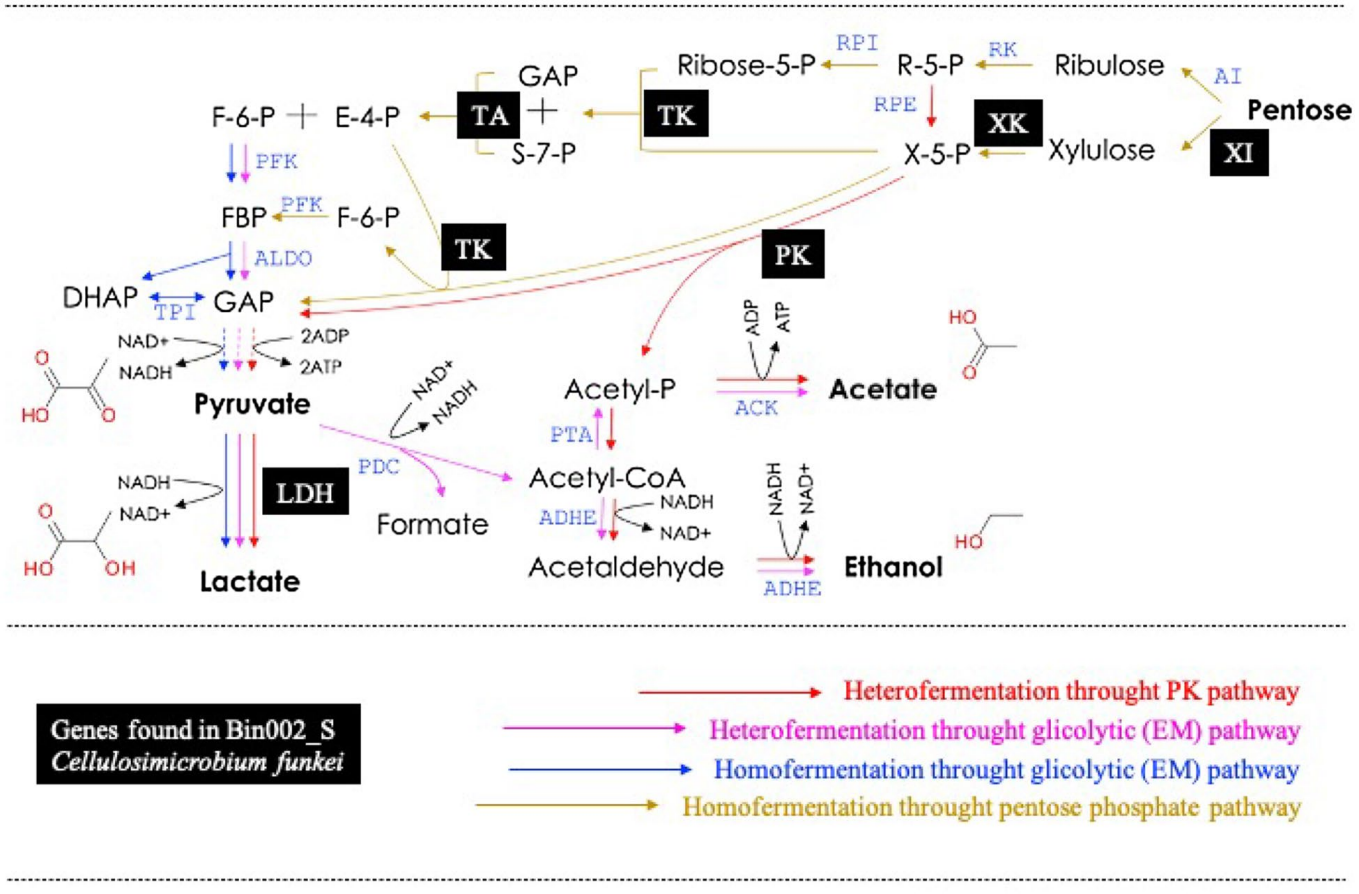
Metabolic pathway for pentoses dissimilation in lactic acid bacteria (modified from Hatti-Kaul et al., 2018). Black squares represent the genes found in the Bin002_S (*Cellulosimicrobium funkei*). XI, xylose isomerase; XK, xylulose kinase; TK, transketolase; TA, transaldolase; PK, phosphoketolase; and LDH, lactate dehydrogenase. For details in all abbreviations see Hatti-Kaul et al. (2018).

A total of five MAGs (Bin6_S, Bin002_S, Bin2_S, Bin4_T10, and Bin44_S) were selected in order to analyze their potential to produce lactic acid from sugars. These MAGs contained at least one LDH encoding-gene (Table 2). Based on the functional annotation using the RAST server, encoding-genes involved in fermentation of pentoses and hexoses were detected within these five MAGs (Supplementary Table S2). The results showed that Bin6_S (*E. casseliflavus*), Bin002_S (*C. funkei*), and Bin2_S (*K. aerogenes*) encode complete enzymatic machinery to produce lactic acid. Specifically, Bin6_S and Bin002_S contain the genes necessary for heterolactic acid fermentation of pentoses (e.g., xylose and arabinose) through the phosphoketolase (PK) pathway (Figure 6). In addition, Bin002_S has the metabolic potential to carry out a homolactic fermentation of xylose through the pentose phosphate (PP) pathway (Figure 6) and heterolactic fermentation of maltose through the PK pathway. Moreover, Bin2_S contains the gene capability to produce D-lactate from glycerol through an Embden–Meyerhof (EM) glycolytic pathway (Supplementary Table S2).

## Discussion

The ensiling of forage crops is a relevant process that enhances feed quality and productivity in livestock systems. The design, application, and testing of starting inoculants is a world-wide priority of research within this field (Muck et al., 2018; Fabiszewska et al., 2019; Nazar et al., 2021a). Thus, this study aimed to build up a unique bioinoculant composed of LAB and LB derived from the phyllosphere of *M. maximum* cv. Agrosavia Sabanera. This Guinea grass is recognized as one of the best species that improves beef and milk production, especially in tropical countries (Villegas et al., 2020; Carvajal-Tapia et al., 2021). In addition, this pasture crop has an excellent nutritional quality and the ability to tolerate adverse abiotic conditions (e.g., drought), being a species of interest for arid regions (Benabderrahim and Elfalleh, 2021). However, the information about its epiphytic microbial community as a source of LAB and LB is null. Ruminal-derived microbial communities could contain higher proportion of LAB and LB. However, we have selected roughage phyllosphere because its microbial communities are the starter inoculant in a silage process.

Previously, the design of consortia composed of lactic acid-producing and lignocellulolytic microorganisms has been achieved by the co-culture of axenic strains (Li et al., 2018; Shahab et al., 2018). In the current work, we developed an innovative top-down strategy to co-cultivate LAB and LB populations using a rational and sequential modification of MRS broth throughout the dilution-to-stimulation approach (Figure 1; Table 1). During the enrichment process, a high abundance of species from *Enterobacteriaceae* family was noticed. Within this enriched system, they could take advantage due to their evolutionary adaptation to the *M. maximum* phyllosphere and also the capacity to grow quickly in liquid environments with labile and plant-derived carbon sources (e.g., glucose; Goldford et al., 2018; Díaz-García et al., 2021). We hypothesized that *Enterobacteriaceae* species could compete with LAB and LB for the uptake of oligo or monosaccharides, having a secondary role in the deconstruction of lignocellulose, where they could be considered as sugar cheaters (Jiménez et al., 2017). In silage, the proliferation of *Enterobacteriaceae* species is undesirable because they compete with LAB, can degrade proteins to produce biogenic amines and branched fatty acids (Ávila and Carvalho, 2019).

Although, the modification of MRS broth did not significantly affect the bacterial composition on a taxonomic basis, the substrate weight loss values (after T6) and the enrichment of some enzyme encoding-genes (i.e., from T2 to T10 and S) suggested that these changes were effective to increase the proportion of LB. However, at the end of the selective process (i.e., T10), a co-existence of LAB (mostly belonged to *Lactobacillales*) and LB (e.g., *Actinomycetales* and *Bacillales*) was observed. In silage, we hypothesized that the action of lignocellulolytic enzymes could improves plant polysaccharides degradation, proving fermentable sugars for the entire microbial community, including LAB. In this regard, the supplementation of cellulases and LAB has shown positive effects in the silage of tropical grasses (Khota et al., 2016). In our enrichment strategy, LAB can grow more slowly because they are metabolically less efficient, but they are competitive and can thrive as a minor fraction of the population with an important ecological role by employing several mechanisms to inhibit the growth of other species (e.g., production of organic acids and hydrogen peroxide; Ávila and Carvalho, 2019). Other mechanism that could back up the coexistence of LB and LAB is the cross-feeding. In this scenario, LB can growth using plant-derived monosaccharides, secreting molecules that LAB might consume (Seth and Taga, 2014).

Many factors, such as nutrient availability, biological interactions, temperature and pH modulate population dynamics in microbial systems (Ratzke and Gore, 2018; Xu et al., 2020; Estrela et al., 2021). In this study, drastic changes in the structure/function of the final selected consortium (i.e., T10) were observed after its growth in a higher volume (500ml) and longer time of incubation (8days; i.e., the scale up growth process). We hypothesized that incubation time, oxygen availability, nutrient content/exchange, and spatial co-localization of the bacterial consortium members (in above mentioned culture conditions) could be factors that drive the populations dynamics in this system (Takahashi and Aoyagi, 2018; Jawed et al., 2019; van Tatenhove-Pel et al., 2021). In addition, these results suggested that our mixed consortium, composed of around 70 bacterial species, is still unstable. A microbial inoculant with less diversity could be more stable (Jiménez et al., 2017). After the scale up growth process, an increase of species from *Paenibacillaceae* family was observed. These types of bacteria could have lower growth rates in a competitive environment and dominate later stages of carbon decomposition (Zhang et al., 2020; Díaz-García et al., 2021). Within silage, *Paenibacillus* species (spore-forming bacteria) could enhance growth of LAB, tolerate acidic conditions and produce bacteriocins that inhibit yeast and molds (Xu et al., 2018; Singh et al., 2019). However, it is important to note that in some cases *Paenibacillus* can compete with LAB for fermentable sugars, limiting its growth (Li et al., 2020). In addition, they have a vast lignocellulolytic potential that may improve the digestion of complex carbohydrates in the bovine rumen (Ticona et al., 2021). *Paenibacillus* species can survive harsh environmental conditions and can be detected even in the last stages of ensiling (Ning et al., 2017). On the other hand, the pH has also modulated the structure, diversity, and metabolic activity of the selected consortium (i.e., T10). In this regard, an increase of lignocellulose consumption and enzymatic activities was observed at pH 5. These results were correlated with a significant increase of *Actinomycetales* and *Lactobacillales* species (Figure 5), suggesting that these taxa could be involved in plant biomass transformation and might be metabolically active in a further ensiling process. It has been reported that species from the phylum *Actinobacteria* are highly abundant in fresh forages (e.g., alfalfa, barley, triticale, and oat), but decrease in abundance during the ensiling processes (Duniere et al., 2017; Hu et al., 2020). Low abundance of *Actinobacteria* and high abundance of *Protobacteria* phylum has been observed in Napier grass silage (Nazar et al., 2021a). Species from *Actinobacteria* generally do not grow below pH 4.5 under microaerophilic conditions, and therefore its growth is unlikely to occur when the pH of the silo stabilizes (Hill et al., 2001). Thus, we presumed that *Actinomycetales* with the capacity to thrive at lower pH values were selected in our mixed consortium.

Based on MAGs annotation, *Paenibacillus*, *Cellulosimicrobium*, and *Sphingomonas* appear as key (hemi)cellulolytic members, while *Cellulosimicrobium*, *Enterococcus*, and *Klebsiella* are the lactic acid producers within the mixed consortium. Their coexistence can be related to the preferential metabolism of different plant-derived hexoses (i.e., glucose or galactose) and pentoses (i.e., xylose and arabinose; Hatti-Kaul et al., 2018). Within the mixed consortium, *Enterococcus* species have the potential to transform xylooligosaccharides and/or arabinoxylans. Although rare, the presence of GH1, GH2, GH3, GH5, GH30, GH43, and GH67 families were found in Bin6_S and Bin3_T10. This information could add new perspectives on the carbohydrate metabolism of LAB species involved in the fermentation of hemicellulose-containing substrates (Michlmayr and Kneifel, 2014). To highlight, we found that *C. funkei* (Bin002_S) has the genomic potential to produce lactic acid from xylose and maltose (through the PK and PP metabolic pathways). Two different pathways are proposed for pentose metabolism, the PK pathway yields 1mol lactic acid/mol sugar, whereas the pentose phosphate pathway provides a theoretical lactic acid yield of 1.67mol/mol sugar (Tanaka et al., 2002). Based on the CAZyme annotation, *C. funkei* could produce enzymes involved in (hemi)cellulose degradation (e.g., GH16, GH30, GH43, and GH51; Ventorino et al., 2015; Dou et al., 2020). To the best of our knowledge, no native (hemi)cellulolytic LAB from *Actinobacteria* phylum has been previously reported. Interestingly, we also found that Bin002_S contained two carbohydrate esterase (CE1) enzymes that may improve the digestibility of forage within the rumen (Li et al., 2021). However, the presence of FAE was not detected within the reconstructed MAGs. Interestingly, *Cellulosimicrobium* and *Paenibacillus* species have been cataloged as desirable microorganisms in cassava foliage silage (Li et al., 2020). Finally, this study reported the design and characterization of a mixed consortium, obtained from the *M. maximus* phyllosphere, composed of LAB and LB. Based on the results, we conclude that a synthetic bioinoculant composed of strains of *Cellulosimicrobium*, *Enterococcus*, and *Paenibacillus* species could be used to improve the quality of silage processes. Further studies with our mixed consortium will: (i) apply it as starting inoculant in an ensiling process and evaluate the consequences in terms of quality and the dynamics of microbial populations; (ii) isolate the key bacterial members; (iii) design a synthetic consortium using bacterial axenic cultures (bottom-up approach) and test it in an ensiling process; and (iv) evaluate lactic acid production by *Cellulosimicrobium via* genome-based metabolic reconstruction using Bin002_S.

## Data Availability Statement

The bacterial 16S rRNA gene amplicon sequencing data obtained in this study have been deposited under NCBI BioProject accession number PRJNA734654. All metagenome sequences are publicly accessible on the MG-RAST server (Metagenome IDs mgm4912842.3 to mgm4912847.3).

## Author Contributions

LD-G carried out all wet-lab experiments and bioinformatic analysis and helped to build up the text draft. DC assisted in all wet-lab experiments. HJ provided the pre-enrichment material. AB, HJ, LG-R, and EB-E contributed to the project design and helped in drafting the manuscript. EB-E coordinated the project and gave financial support. DJ coordinated and conceived the project and drafted the manuscript. All authors contributed to the article and approved the submitted version.

## Funding

This work was supported by the FAPA project (number PR.3.2018.5287) obtained by DJ at the Universidad de los Andes (Bogotá, Colombia) and a granted project (Number EXT-2019-64-1677) obtained within the cooperation Agrosavia-UniAndes. All authors acknowledge financial support provided by the Vice Presidency for Research and Creation at the Universidad de los Andes.

## Conflict of Interest

The authors declare that the research was conducted in the absence of any commercial or financial relationships that could be construed as a potential conflict of interest.

## Publisher’s Note

All claims expressed in this article are solely those of the authors and do not necessarily represent those of their affiliated organizations, or those of the publisher, the editors and the reviewers. Any product that may be evaluated in this article, or claim that may be made by its manufacturer, is not guaranteed or endorsed by the publisher.

## Supplementary Material

Supplementary Figure S1 Relative proportion (%) of the most common lactic acid bacteria (LAB) found in silage processes. Metagenomic sequence taxonomic assignment was carried out by using RefSeq database.

Supplementary Figure S2 Secreted endo-enzymatic activities (OD values) on **(A)** six chromogenic polysaccharide hydrogels (CPH) and **(B)** two insoluble biomass substrates (ICB) after T10 consortium growth on unmodified pH 6.2, pH 5, and pH 4.

Supplementary Figure S3 Relative proportion (16S rRNA data) of taxa (class) after incubation of the consortium at T10 in a range of pH values. Error bars represent SDs from three biological replicates.

Supplementary Table S1 Detailed features of metagenome assembled genomes (MAGs) reconstructed from T2, T10, and S consortia.

Supplementary Table S2 Number of genes involved in lactic acid production found within Bin6_S, Bin002_S, Bin2_S, Bin4_T10, and Bin44_S.
