# Supplementary material for: Top-Down Enrichment Strategy to Co-cultivate Lactic Acid and Lignocellulolytic Bacteria From the Megathyrsus maximus Phyllosphere
Source: Front Microbiol. 2021 Nov 2;12:744075. doi: 10.3389/fmicb.2021.744075 (PMC8753987; doi:10.3389/fmicb.2021.744075)
Supplement: Supplementary Figure S1 — Relative proportion (%) of the most common lactic acid bacteria (LAB) found in silage processes. Metagenomic sequence taxonomic assignment was carried out by using RefSeq database. [file Presentation_1.pptx]

## Slide 1
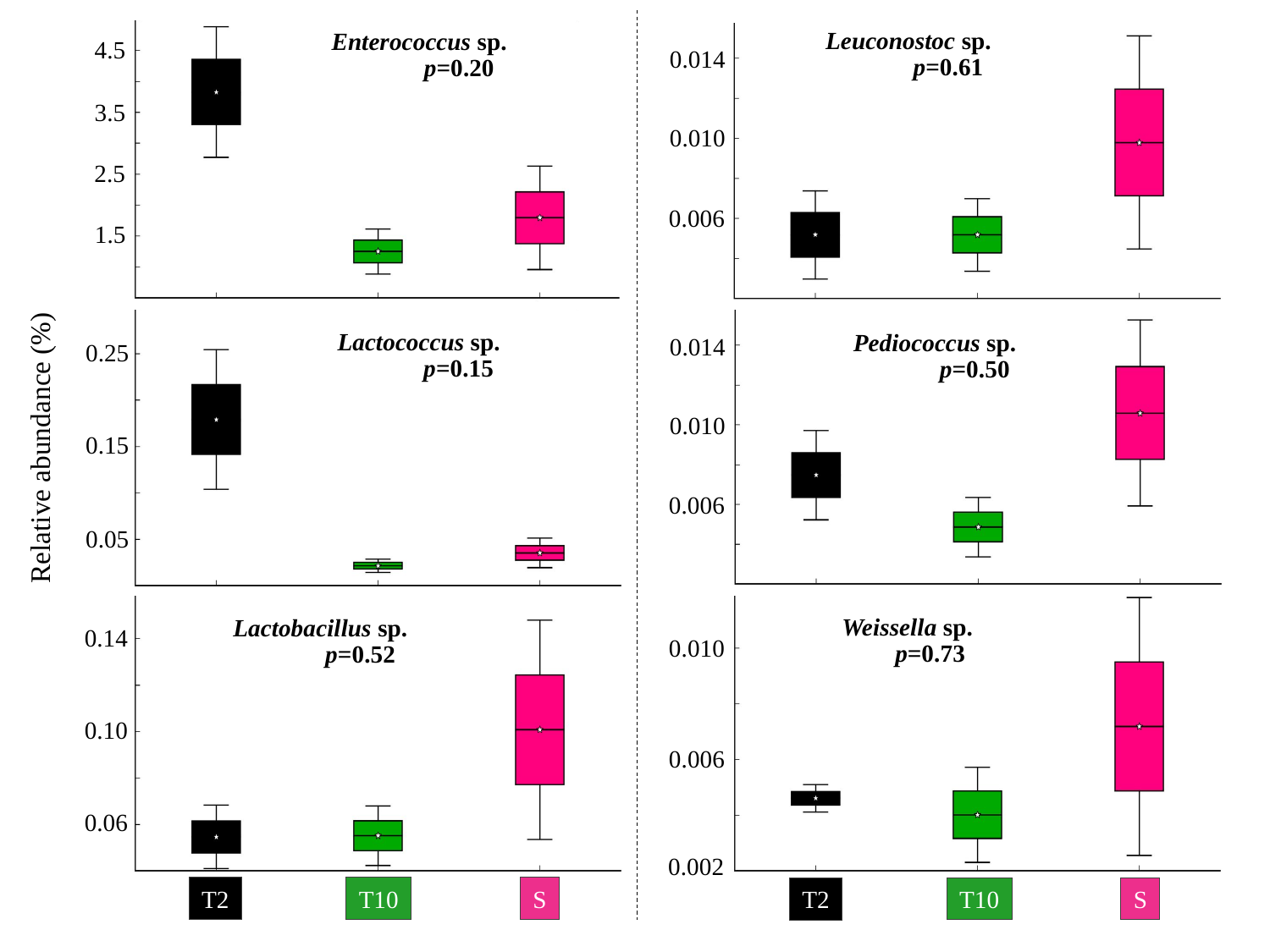

Leuconostoc sp.
Enterococcus sp.
4.5
0.014
p=0.61
p=0.20
3.5
0.010
2.5
0.006
1.5
Lactococcus sp.
Pediococcus sp.
0.014
0.25
p=0.15
p=0.50
0.010
0.15
Relative abundance (%)
0.006
0.05
Weissella sp.
Lactobacillus sp.
0.14
0.010
p=0.73
p=0.52
0.10
0.006
0.06
0.002
T10
S
T2
T10
S
T2

## Slide 2
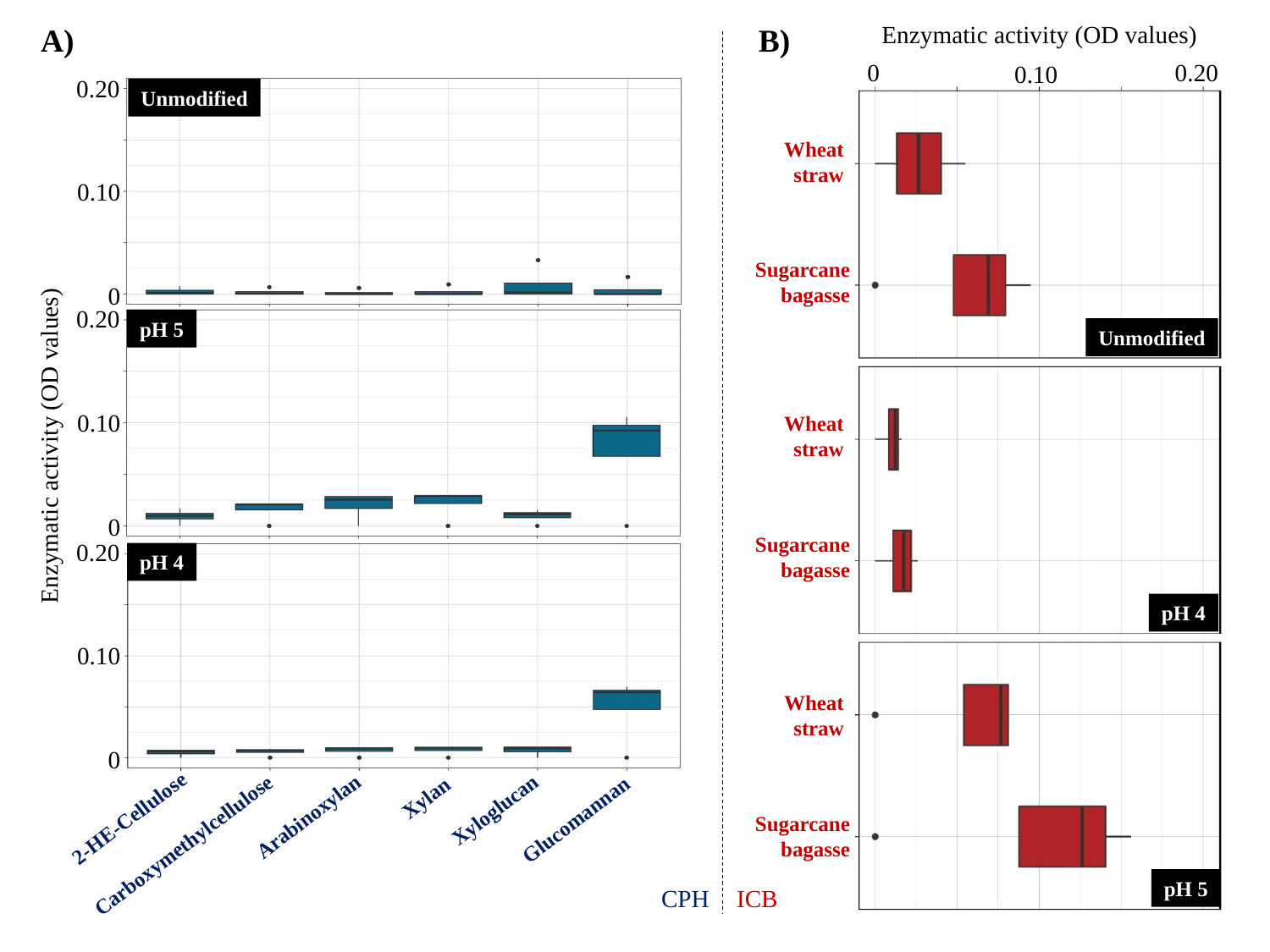

Enzymatic activity (OD values)
A)
B)
0
0.20
0.10
0.20
Unmodified
Wheat
straw
0.10
Sugarcane
bagasse
0
0.20
pH 5
Unmodified
0.10
Wheat
straw
Enzymatic activity (OD values)
0
Sugarcane
bagasse
0.20
pH 4
pH 4
0.10
Wheat
straw
0
Xylan
Xyloglucan
Arabinoxylan
2-HE-Cellulose
Glucomannan
Sugarcane
bagasse
Carboxymethylcellulose
pH 5
CPH
ICB

## Slide 3
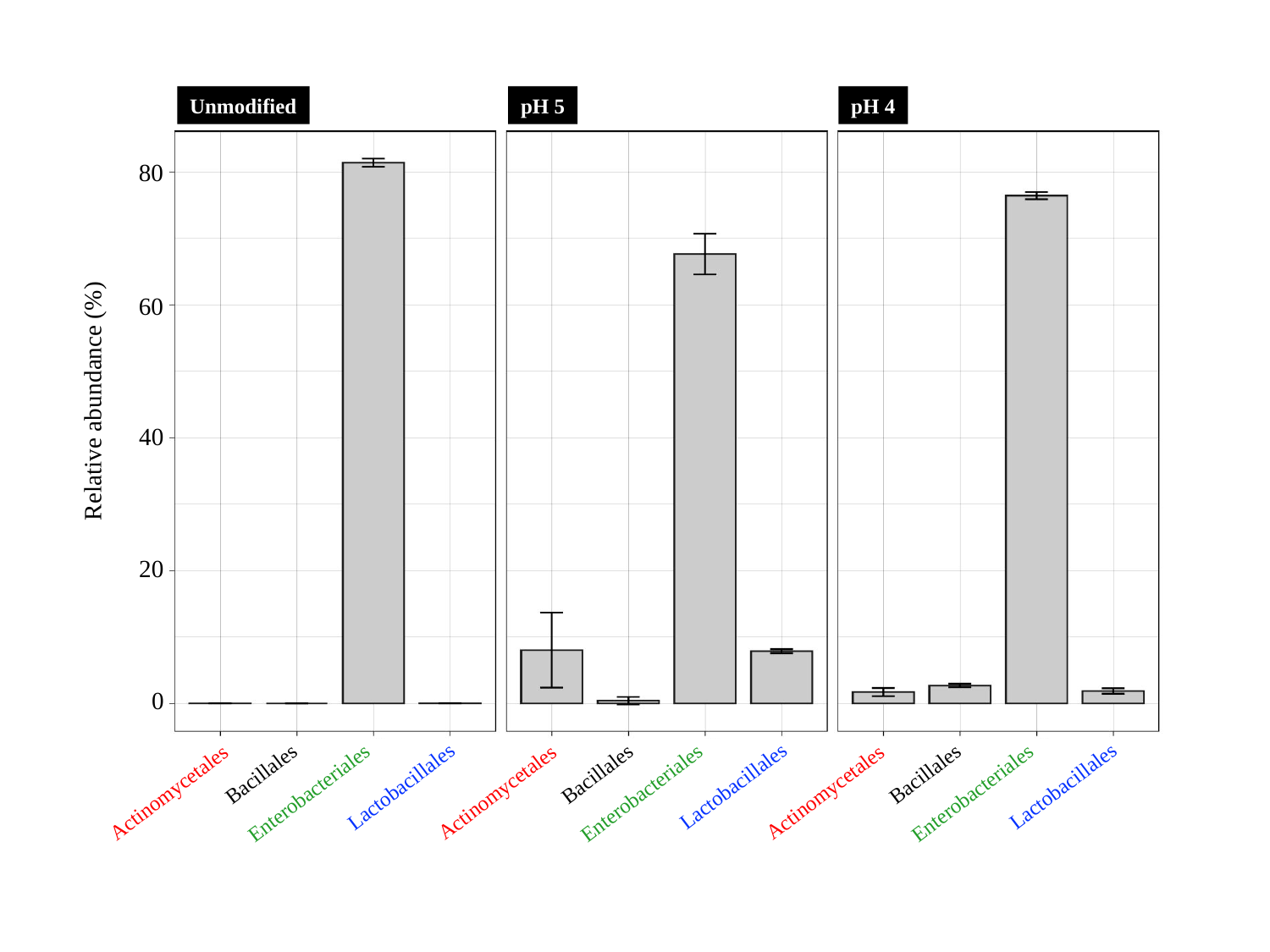

pH 4
pH 5
Unmodified
80
60
Relative abundance (%)
40
20
0
Bacillales
Bacillales
Bacillales
Lactobacillales
Lactobacillales
Lactobacillales
Actinomycetales
Actinomycetales
Actinomycetales
Enterobacteriales
Enterobacteriales
Enterobacteriales
